# Investigating Demographic Barriers Toward Virtual Reality Pain Efficacy in Clinical Trials: An Exploratory Mixed-Methods Study

**DOI:** 10.1177/29941520251378444

**Published:** 2025-12-29

**Authors:** Omer Liran, Allistair Clark, Zoe Krut, Sam Eberlein

**Affiliations:** Cedars-Sinai Medical Center, Los Angeles, California, USA.

**Keywords:** chronic pain, digital divide, diversity, medical extended reality, mixed-methods research, virtual reality

## Abstract

Chronic pain affects over 50 million Americans and is frequently managed with pharmacologic interventions, including opioids, which carry misuse risks. Virtual reality (VR) has emerged as a promising non-pharmacological therapy, yet questions persist about equitable access and whether VR could perpetuate existing health care disparities. This exploratory sequential mixed-methods study investigated barriers and facilitators to VR therapy use in diverse chronic pain populations and examined whether these challenges correlated with changes in Patient-Reported Outcomes Measurement Information System (PROMIS) Pain Interference (PROMIS-PI) scores. We recruited 31 participants from two National Institutes of Health-funded trials, utilizing purposive sampling to ensure a broad demographic range regarding race, ethnicity, gender, socioeconomic status, and geographic location. Participants completed one-on-one interviews regarding VR use, which were qualitatively coded and quantified to explore correlations with changes in PROMIS-PI from baseline to week 8. Six themes emerged from the qualitative analysis: apprehension and familiarity, usability and comfort, application and pain, experiences, personal connection, and community beliefs. Most participants reported at least temporary pain relief with VR, citing distraction-based or mindfulness content as beneficial; however, cost, technological literacy, and cultural familiarity were identified as key barriers. Spearman’s $\rho$ and point-biserial tests showed no statistically significant relationships between thematic variables or demographics and PROMIS-PI changes. These findings suggest that VR therapy is broadly acceptable and beneficial across diverse patient groups, though cost and cultural apprehension may impede widespread, equitable adoption. Future research should focus on refining VR content for user comfort, expanding technological support, and developing strategies to overcome barriers to access to ensure the therapy’s benefits are realized equitably.

## Introduction

Chronic pain, defined by the International Association for the Study of Pain as pain that persists or recurs for longer than 3 months, is a debilitating condition that affects over 50 million Americans and accounts for approximately one in five visits to primary care providers.^1^ Defined as pain persisting for 3 months or longer, it not only causes physical distress but also impacts emotional and social well-being, often leading to a markedly reduced quality of life.^2^ Traditional pain management strategies rely heavily on pharmacological approaches, including opioid analgesics, which carry risks of adverse effects, misuse, dependence, tolerance, and overdose.^3,4^ The opioid epidemic, fueled by the overprescription of opioid medications for chronic pain, has had a devastating social and economic impact. In 2020 alone, the economic toll of the opioid crisis in the United States was estimated to be nearly $1.5 trillion, and it has disproportionately affected marginalized communities. In response to escalating opioid use, federal efforts—such as the National Institutes of Health (NIH) Helping to End Addiction Long-term Initiative—have prioritized both improved treatments for opioid addiction and the exploration of non-pharmacological pain management interventions.^5^

Virtual reality (VR) therapy has emerged as a promising, non-pharmacological alternative for pain management. Previous studies have demonstrated the effectiveness of VR in reducing pain in various clinical settings. For example, a randomized controlled trial found that hospitalized patients who used a VR program experienced a significant reduction in pain scores compared with a control group.^6^ The study also found that the effects of VR were independent of the cause of pain, suggesting broad applicability. Another study showed that repeated VR sessions provided significant and lasting pain relief for individuals with chronic neuropathic pain, with some participants reporting a complete cessation of pain during VR sessions. These studies, among others, have shown that VR can be an effective tool for pain management, though the specific mechanisms of action are still being investigated. It is believed that VR works by distracting the user from pain and by modulating the user’s emotional and cognitive experience of pain.

Further recent trials have investigated VR specifically for common chronic pain conditions such as chronic low back pain (cLBP), with a study demonstrating meaningful reductions in pain interference using a VR-based therapeutic (EaseVRx, AppliedVR).^7^ In parallel, agencies such as the National Institute of Arthritis and Musculoskeletal and Skin Diseases (NIAMS) and the National Institute of Nursing Research have funded research to evaluate VR for chronic pain in diverse settings—including rural communities—highlighting its growing importance.^8,9^ Nevertheless, as digital therapeutics expand, concerns regarding equitable access persist. Black and Hispanic patients, older adults, and individuals in rural or low-socioeconomic status environments frequently face limited access to digital resources, raising the possibility that investment into VR intervention development could further exacerbate rather than alleviate health care disparities.^10–12^

Effective implementation of VR therapy thus requires not only evidence of clinical efficacy but also a thorough understanding of the barriers and facilitators that shape its use, particularly among marginalized groups. These barriers may include limited technology access, differing levels of digital literacy, cultural or social norms, and discomfort or reluctance related to wearing a head-mounted display.^10–12^ The extent to which such factors are associated with clinical outcomes, however, remains largely unknown.

Building on two parent randomized trials examining VR therapy for both cLBP and broader chronic pain populations,^7,8^ we conducted an exploratory sequential mixed-methods study. Our objectives were to (1) identify the technological and cultural barriers encountered by participants using VR, (2) investigate whether these reported barriers correlated with changes in PROMIS Pain Interference (PROMIS-PI) scores, and (3) explore how demographic characteristics might influence these reported barriers. Ultimately, we aimed to guide the design of future VR-based pain management interventions that are not only effective but also feasible and acceptable for diverse patient populations.

Our qualitative research questions were as follows:

1. What challenges did participants face when utilizing the active VR treatment for chronic pain?
2. Did aspects of the intervention, shaped by participants’ lived or cultural experiences, encourage continued engagement?

Subsequently, our mixed-methods research questions were as follows:

1. Do differences in reported challenges explain variations in PROMIS-PI score changes from baseline to week 8 in the parent studies?
2. How do differences in reported challenges relate to demographic variations among those who reported them?

By integrating both qualitative and quantitative perspectives, our study sought to provide a comprehensive evaluation of the potential therapeutic impact of VR therapy across diverse populations while also highlighting any obstacles that may impede its widespread adoption. Addressing these issues proactively could foster more equitable and successful incorporation of VR into standard care for chronic pain management.

## Methods

### Design description

This mixed-methods investigation used an exploratory sequential design. Following Morse’s notation (QUAL → quan), the study’s primary data collection consisted of one-on-one, semi-structured interviews with participants selected from two NIH-funded trials (CT.gov IDs: NCT04409353 and NCT04933474).^13^ The qualitative data from these interviews were then quantized to analyze differences in PROMIS-PI change scores and demographic data.

### Rationale

An exploratory sequential mixed-methods approach was chosen because the parent trials had relatively narrow quantitative objectives and optional-response usability data. Qualitative inquiry allowed participants to articulate common challenges to treatment utilization in their own words, providing a more comprehensive perspective on the range of difficulties encountered. By subsequently quantizing these qualitative findings, we sought to ascertain how these challenges might influence treatment effectiveness. Thus, this approach enabled a nuanced understanding of both the types of barriers experienced and their potential quantitative associations with clinical outcomes.

### Participants

A power analysis for Spearman’s ρ correlation informed the target sample size of 31 participants, each previously enrolled in the active VR intervention arms of the two recent randomized controlled trials. A purposeful sampling strategy balanced representation by race, gender, ethnicity, age, and geographic location (rural versus non-rural). Participants were selected from the recruitment logs respective to the previous clinical trial that they had completed participation in. A demographically balanced oversample of potential participants was created prior to consent. We then contacted selected patients by phone, in which we discussed the aims of this supplemental investigation and obtained consent. If the patient consented, their email was retrieved from the original recruitment log and an appointment for a single virtual meeting. This heterogeneous sample both approximated the demographic composition of the parent trials and fostered maximum variation in experiences (Table 1).

**Table 1.** Demographics

Total: *N* = 31	*n*	%
Gender		
Male	11	35.48
Female	20	64.51
Race		
Non-Hispanic White	13	41.93
Hispanic White	5	16.13
Black or African American	7	22.58
Asian	2	6.45
Indigenous Americans and Alaskan Natives	1	3.22
Other	3	9.67
Ethnicity		
Hispanic	11	35.48
Non-Hispanic	18	58.06
Other	2	6.45
Rural status		
Rural	15	48.39
Not rural	16	51.61
Education status		
Some high school	2	6.45
High school/GED	9	29.03
Some college completed	2	6.45
Associate’s/technical degree	3	9.67
Bachelor’s degree	6	19.35
Graduate degree	6	19.35
Doctoral/postgraduate	2	6.45
Vocational/trade school	1	3.22
Income		
<$10,000	1	3.22
$10,000–$24,999	6	19.35
$25,000–$34,999	1	3.22
$35,000–$49,999	5	16.13
$50,000–$74,999	4	12.90
$75,000–$99,999	2	6.45
$100,000–$149,999	1	3.22
$150,000–$199,999	4	12.90
≥$200,000	2	6.45
Prefer not to answer	5	16.13
	Minimum–maximum (range)	Mean (standard deviation)
Age	36–87 (51)	57.81 (11.64)

GED, General Equivalency Diploma.

The two clinical trials were chosen for participant recruitment sources due to their utilization of the same active VR intervention, the arm from which all participants were recruited, and their similar goals in addressing chronic pain symptomology. The first trial, NCT04409353, was a randomized double-blind, placebo-controlled trial that aimed to compare the efficacy of the active VR intervention against a distraction VR and sham VR arms in managing cLBP. The second trial, NCT04933474, was a two-arm, multi-center, virtual randomized controlled trial that aimed to compare the effectiveness of the active VR intervention against a non-VR digital pain management program for managing general chronic pain. Both trials were included as sources of recruitment as the latter trial encompassed a primarily rural population, whereas the former a primarily urban population. This allowed us to explore the possible differences in VR utilization and experience between populations with potentially disparate accessibilities and exposure to technology.

### Data collection

After providing informed consent, participants completed a single, one-on-one semi-structured interview about their experiences using the active VR intervention. The interview protocol, jointly developed by three trained study staff, explored preferences, physical comfort, technology-related frustrations, and cultural or lived background resonance with the program (see the interview protocol in the Supplementary Data). Because the sample size was determined by statistical power, the concept of thematic saturation was not employed.

Quantitative data were extracted from existing records and included participant demographics (age, race, ethnicity, gender, education level, household income, and geographic living location) and PROMIS-PI change scores from baseline to week 8 of the parent studies. Week 8 of PROMIS-PI data collection was used as the active VR intervention software, followed by an 8-week use program.

### Data analysis

Qualitative analysis followed a content analytic approach with multiple coding passes. One qualitative expert, who also conducted the interviews, performed iterative coding to refine codes and create overarching code categories:

1. Open coding (first pass): Codes emerged organically from “poignant” participant statements.
2. Focused coding (second pass): First-level codes were grouped into nascent categories at natural pauses, typically after every five transcripts.
3. Focused coding (third pass): Nascent categories were further refined into a final set of six thematic categories.

Next, these six categories were transformed into quantitative variables. Dichotomous variables were used to indicate the presence (1) or absence (0) of explicit statements relevant to each category. In some cases, nominal variables distinguished subcategories (e.g., different types of usability issues). Regarding such nominal variables, if a participant neither explicitly through direct response to the interview protocol nor implicitly by addressing the topic in natural conversation confirmed nor refuted a particular category, it was treated as missing data. In this case, the participant’s response option was marked as missing.

Following quantization, Spearman’s ρ correlations (and point-biserial correlations for sensitivity analyses) assessed the relationships between these variables and PROMIS-PI changes from scores at baseline to week 8 in the parent trials. *p* Values generated from Spearman’s ρ correlations were reported both due to the non-normality of PROMIS-PI data and due to the categorical and bivariate nature of generated quantitative codes. Lastly, chi-square tests (for nominal demographic data) and independent-samples *t*-tests (for age data) examined potential relationships between demographic characteristics and each quantized variable.

## Results

### Participant characteristics

**FIG. 1.**
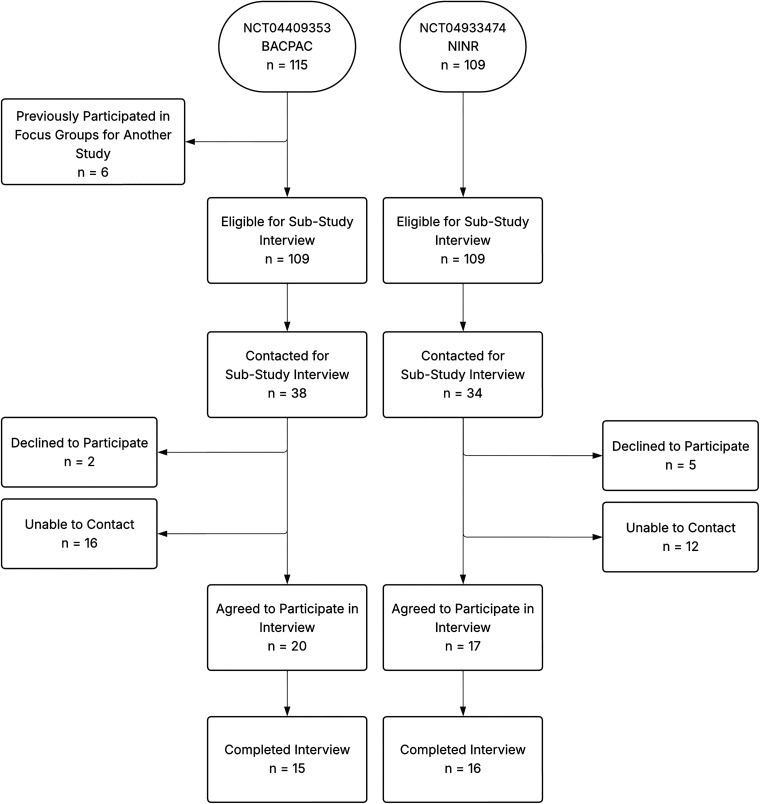
Recruitment process of this investigation from start (top) to finish (bottom) of the investigation.

Figure 1 provides a full breakdown of the recruitment process for this investigation. A total of 31 participants were recruited from the two previous trials. Of these, 20 (65%) were male, and 13 (41%) identified as White non-Hispanic, with a mean age of approximately 57.8 years. Table 1 provides the full demographic breakdown.

### Qualitative findings

Six thematic categories emerged from the 31 participant interviews, capturing explicit barriers to using the intervention as well as factors that either enhanced or hindered the overall experience. See Supplementary Table S1 in the Supplementary Data for exemplary quotations corresponding to each emergent theme.

#### Participation apprehension and familiarity

Many participants were driven to enroll because of high pain levels, with some “willing to try anything” and others hoping for a solution that “wasn’t… invasive or surgical in nature or medication related….” Initial uncertainty about VR was common, including not knowing “what the program looked like” or feeling “afraid I was going to break the [device]….”

For those with no prior VR experience, responses ranged from trepidation to “excitement.” Among participants who had used VR previously, none had “utilized [VR] in a form where it could actually be beneficial,” though they felt somewhat more prepared for the technology. Twelve participants (38.71%) reported prior VR experience, compared with 19 (61.29%) without any. Those with prior experience tended to have higher median incomes tended to have higher median incomes, and prior VR experience was more common among males and Hispanic participants.

#### Usability and comfort

Learning to operate the headset was described as “quick” or “easy” by 14 participants (45%), with some stating, “…once I got the hang of it. It was very easy,” thanks to “directions [being] really basic…” and an ability to learn through trial and error. Common issues included in-program vision orientation (13%) and tracking user progress through the program (13%).

Regarding comfort, 11 participants (35.48%) reported no discomfort. Others found the headset weight problematic or “heavy,” with nine participants (29%) reporting discomfort, including exacerbation of neck pain: “…in my neck… It is heavy.” Among the 12 participants (39%) who reported usability issues, there was a younger median age and a higher proportion of Black or African American individuals, males, and higher-income earners compared with those who did not report such issues.

#### Application and pain

Fifteen participants (48.38%) noted a clear reduction in pain: “[it] really did help me with my chronic pain in my back,” with 5 (16.13%) indicating a permanent reduction and 10 (32.26%) experiencing relief only during active VR use. One participant (3.23%) reported no effect on pain. Those who perceived a pain reduction had a slightly higher median income and tended to have lower educational attainment compared with those who reported no change.

#### Experiences

Participants chose among mindfulness exercises, games, or nature simulations based on personal pain management strategies. Distraction was key for some: “The scenery and the sounds… were distracting.” Others focused on the interplay between mood and pain, appreciating the stress relief offered by quieter experiences. Mindfulness exercises taught skills such as breathing techniques that helped outside of VR, and nature simulations offered immersive calm. In contrast, some avoided “high engagement” activities such as games due to added stress, while others found games most effective for making them “forget my pain.”

Of the 31 participants, 21 (67.74%) identified a single preferred experience, which fell into three categories:

1. Mindfulness experiences (guided meditation or breathing exercises),
2. Games (interactive experiences for distraction and entertainment), and
3. Nature experiences (3D audiovisual simulations of outdoor environments).

#### Personal connection

Fourteen participants (45.16%) felt a direct link between their personal background and how they engaged with VR. Those who enjoyed nature scenes often cited relevant life experiences—“I am a diver … it felt like I was back doing that.” Game enthusiasts often described themselves as adventurous or competitive: “I love winning … so that’s why I like to do it long and do it focused.” Technological familiarity also emerged: “I was kind of used to [VR] … I knew what to expect.”

#### Community beliefs

When asked about the hardware and software’s potential accessibility, eight (25.8%) felt it would be feasible for others in their community, while two (6.45%) believed it would not. Many identified possible barriers, including for older adults who “might be a little afraid or concerned” due to unfamiliarity with technology. Cost was also considered prohibitive by some, who reasoned that many “wouldn’t spend that money … if they didn’t know whether it would help.”

### Quantitative results

From these six qualitative categories, four were transformed into binary variables:

1. Prior VR experience (1 = yes, 0 = no),
2. Usability issue (1 = yes, 0 = no),
3. Belief in VR’s efficacy for pain reduction (1 = yes, 0 = no), and
4. Favored experience type (1 = games, 0 = relaxation-based experiences [mindfulness or nature]).

Spearman’s ρ and point-biserial correlations were used to assess relationships between these variables and participants’ PROMIS-PI change scores from baseline to week 8. No statistically significant relationships were found (Table 2). Additionally, a Brown–Forsythe test showed no significant difference in PROMIS-PI change scores between participants favoring games versus those favoring mindfulness or nature experiences.

**Table 2.** Spearman’s ***ρ*** and Point-Biserial Correlations Between Quantized Variables and Delta PROMIS Pain Interference Scores

Variable	Spearman’s ***ρ***	*p*	Point-biserial	*p*
Prior VR experience	−0.257	0.170	−0.310	0.095
Usability issue presence	−0.162	0.392	−0.122	0.519
Belief in intervention efficacy	0.012	0.951	0.056	0.768

VR, virtual reality.

Subsequent chi-square tests using these four quantized variables yielded no significant associations with sex, race, ethnicity, rural/non-rural living location, highest education level, or household income (Table 3). After confirming normality, multiple *t*-tests revealed no statistically significant relationships between age and any of the four variables (Table 4).

**Table 3.** Pearson’s Chi-Squared Test Between Quantized Variables and Demographics [**χ**^2^ (*df*)]

	Sex	Race	Ethnicity	Rural status	Education	Income
Prior VR experience	0.92 (1)	2.78 (6)	4.28 (3)	0.93 (1)	14.14 (10)	10.49 (8)
Usability issue presence	0.034 (1)	3.84 (6)	2.24 (3)	0 (1)	6.41 (10)	10.54 (8)
Belief in efficacy	0.00 (1)	7.53 (6)	2.28 (3)	0 (1)	11.65 (10)	6.20 (8)
Favored experience	0.04 (1)	2.55 (4)	0.39 (2)	0.23 (1)	7.29 (9)	4.35 (7)

* All analyses of demographic variables yielded a *p*-value > 0.05.

**Table 4.** Independent tests Between Quantized Variables and Participant Age

Quantized variable	*T* (*df*)	*p*
Prior VR experience	−0.72 (22.64)	0.476
Usability issue presence	2.00 (28.43)	0.054
Belief in efficacy	1.23 (27.02)	0.229
Favored experience	−0.60 (13.08)	0.555

## Discussion

In this mixed-methods investigation of VR therapy for chronic pain, we examined user perspectives from a demographically diverse cohort and quantified whether specific barriers or facilitators were associated with changes in PROMIS-PI scores. Our findings suggest that, overall, VR therapy is acceptable and shows promise for pain management across a broad range of socioeconomic, geographic, and racial/ethnic backgrounds. Yet, several important considerations emerged concerning technological apprehension, perceived cost, cultural familiarity, and sustainability of pain relief.

### Interpretation of key findings

Consistent with existing literature, many participants reported at least temporary pain relief while engaged with VR. These benefits were often attributed to distraction and mindfulness features, echoing earlier studies describing VR’s capacity to shift cognitive focus away from pain. Notably, although participants across different demographic groups reported varying degrees of apprehension and different preferences in content (e.g., mindfulness versus games), there was no statistically significant correlation between any of these thematic variables (e.g., prior VR experience, usability challenges) and changes in PROMIS-PI scores. This finding implies that, within this relatively small sample, usability issues or prior familiarity with VR did not appear to systematically influence clinical outcomes.

Interestingly, participants who had used VR before did not universally find it easier to adopt the study’s headset or software, suggesting that general familiarity with VR-based entertainment may not directly translate to reduced barriers in a therapeutic context. Similarly, while cost and cultural unfamiliarity emerged as concerns, their impact on short-term pain outcomes was not clearly discernible from our data. These issues, however, remain critically important for the equitable dissemination of VR therapies in real-world settings.

### Equity and accessibility considerations

Our qualitative data reinforce that cost and community-level beliefs about VR’s usefulness can pose significant barriers to widespread implementation, particularly among underserved groups. Older adults, individuals with lower technological literacy, and those with limited financial resources may be hesitant to invest time or money into a VR program unless there is strong evidence of efficacy and a user-friendly learning curve. Although the lack of significant correlations with demographic variables is somewhat reassuring, it does not negate the potential for VR to exacerbate health care disparities if devices remain unaffordable or technologically complex. Future research and policy efforts should focus on subsidizing or otherwise reducing the financial burden of VR hardware, alongside designing culturally tailored programs and robust technical support services.

### Limitations

Several limitations must be acknowledged. First, our study is exploratory and may be underpowered to detect subtle correlations between thematic variables and PROMIS-PI scores, especially given the relatively small sample size (*N* = 31). Second, these findings are drawn from participants in two specific NIH-funded trials; their experiences may not fully reflect broader clinical populations or different chronic pain conditions. Third, the decision to “quantize” qualitative themes into binary variables can obscure nuanced or overlapping experiences. Finally, we did not assess whether improvements in pain interference persisted after participants discontinued VR use, so further work is needed to clarify long-term effects.

### Future directions

Future investigations should employ larger, more diverse cohorts to verify whether demographic or usability factors truly remain independent of pain-related outcomes. Trials that offer multiple VR content modalities in a randomized manner, paired with rigorous implementation frameworks such as RE-AIM (Reach, Effectiveness, Adoption, Implementation, and Maintenance),^14^ would help determine how best to scale VR therapy in clinical settings. Moreover, community-based participatory research may illuminate how cultural values and social norms shape acceptance, thus guiding more targeted adaptation of VR programs.

## Conclusions

Our results underscore VR’s potential to provide meaningful, non-pharmacological relief for individuals with chronic pain, with few reported differences in pain outcomes attributable to participants’ background or prior VR experience. Nonetheless, pragmatic barriers—including affordability, cultural skepticism, and technology apprehension—must be proactively addressed to ensure equitable access. By integrating user feedback into program design, establishing robust technical support, and developing cost-containment strategies, VR could become a mainstream, culturally competent modality for safe and effective chronic pain management.

## Authors’ Contributions

O.L.: Conceptualization. O.L., A.C., Z.K., and S.E.: Methodology. S.E., Z.K., and A.C.: Investigation. A.C.: Formal analysis. O.L. and A.C.: Writing—original draft. O.L., A.C., Z.K., and S.E.: Writing—review and editing. O.L.: Funding acquisition.

## Supplemental Material

sj-docx-1-mxr-10.1177_29941520251378444 – Supplemental material for Investigating Demographic Barriers Toward Virtual Reality Pain Efficacy in Clinical Trials: An Exploratory Mixed-Methods Study Supplemental material, sj-docx-1-mxr-10.1177_29941520251378444 for Investigating Demographic Barriers Toward Virtual Reality Pain Efficacy in Clinical Trials: An Exploratory Mixed-Methods Study by Omer Liran, Allistair Clark, Zoe Krut, and Sam Eberlein

## Abbreviations

cLBP: Chronic low back pain
GED: General Educational Development
HEAL: Helping to End Addiction Long-term
IASP: International Association for the Study of Pain
IRB: Institutional Review Board
NIAMS: National Institute of Arthritis and Musculoskeletal and Skin Diseases
NIH: National Institutes of Health
PROMIS: Patient-Reported Outcomes Measurement Information System
PROMIS-PI: PROMIS Pain Interference
QUAL: Qualitative
quan: Quantitative
RE-AIM: Reach, Effectiveness, Adoption, Implementation, and Maintenance
VR: Virtual reality
