# Supplementary material for: Investigating Demographic Barriers Toward Virtual Reality Pain Efficacy in Clinical Trials: An Exploratory Mixed-Methods Study
Source: J Med Ext Real. 2025 Dec 29;2(1):362–70. doi: 10.1177/29941520251378444 (PMC13393001; doi:10.1177/29941520251378444)
Supplement: Supplementary file 1 [file sj-docx-1-mxr-10.1177_29941520251378444.docx]

**Table 1** Themes and corresponding example quotations

| Themes | Quotations |
| --- | --- |
| Participation Apprehension and Familiarity | “…And I know that Video game technology and video VR technology has advanced drastically just in the past, you know, decade. And so I knew that many of those kinds of things are very intense and things like that. So I really didn't know what I would be seeing or doing.”  “I was just kinda excited to see what it was gonna be like, you know. I wasn't apprehensive… just kinda eager, you know. eager to try it.”  “I didn't have any. I just went in there free-minded and said, Hey, you know I'll give it a try. Anything. I've been trying everything else with my back pain and you know. So I was like open mind and say, you know, we'll get it hurt. It's not anything physical. So yeah.” |
| Usability and Comfort | “Yes, the instructions on how much time I was in the VR, how to get to the next level, do I need to go into the settings? It really didn't give me a step-by-step. I had to figure it out myself at some times.”  “It was pretty easy to me. I mean it. I mean you had to… you know, work with it a little bit, and see… where I find this, where I find that. But it wasn't. It was easy to me, you know.”  “[The VR Headset] would hurt my nose, though the you know the thing that goes across the bridge of your nose that would hurt, and I think that's why I stopped using it as much toward the end.” |
| Application and Pain | “I suffer with chronic pain, and I was hoping that the study would have helped me, and it did.”  “I knew I was actively doing something that would benefit me”  “Yes, I found it very helpful managing my pain and I didn't have to take any pain medicine” |
| Experiences | “So it was kind of cool, it really is. It did take your mind off things. And what was kind of interesting was the games I found that to be the most benefit. Because I was like, okay, cause you were so focused on… I'm gonna shoot, shoot this ball and stuff like that. It really did distract you from the pain.”  “[It gave] another way of relaxing where the pain can be taken away through meditation, or even yoga.”  “Uh, the game affected my senses, uh, it heightened my senses where I was more in a, in an alert mode, more of a panic mode. Um, it did take my mind off the pain a little bit, very little, but because of the mode that put me in a panic mode, was I playing the game right, uh, it didn't help break out the pain.” |
| Personal Connection | “Well, I'm from the country. and so I really enjoyed the ones where you feel like you're out in the country, in nature. because it felt like I was home.”  “Um, it seemed a little, I mean, I'm 64, almost 65. So I remember all of those, um, you know, the original headphones that everyone wore, so it was very reminiscent of that. Um, I didn't find [the headset] particularly, you know, it, it, they didn't bother me, but they were very reminiscent of our first headphones, a little clunky, but you know, sort of retro clunky, I guess.”  “I used to live by the ocean, so yeah, it did remind me of when I was a kid and playing in the waters, and it did make me think about the past a little bit, and it was quite enjoyable. Very good program.” |
| Community Beliefs | “Well, like the people who live around me right now are mostly old ladies in their 80 s. And 90 s. So I don't think they would know what to do with it.”  “Some of [my community], you know, if there, if yeah decision on I I mean I don't know the cost, I think would be our headsets like $500, like if you're going out and just buying one. No, a lot of them wouldn't spend that money, especially on something they didn't know whether it would help them or not.”  “Yeah, I think now it is. It was a while back. It was a time, and I would say, no, but now it's getting more and there they're advancing now they're especially with the. You know, Internet has free Internet coming into play a lot better where they can. you know, and and get into the games or get into the virtual reality, and stuff like that is a lot more accessible to them than it will use to be.” |

**Semi-Structured Interview Guide**

1. Before this study, how familiar were you with Virtual Reality, or VR for short?

- What emotions or feelings did these prior experiences evoke?

2. When you first agreed to participate in the study, what were your expectations?

- What personal experiences or beliefs shaped these expectations?

3. I'd love to understand more about your relationship with the VR device:

a) Overall, what are your general thoughts about the VR headset?

b) Who do you think VR, in general, is made for?

c) Describe the first moment of wearing the headset.

- What emotions did it bring up?

- What discomfort, if any, did you encounter during or after prolonged use?

- How would you compare its physical comfort to its emotional impact?

d) Talk me through your typical session.

- What factors influenced how long you spent in the session?

e) Describe your journey of familiarizing yourself with the headset.

- Was using the headset easy to learn (for example, turning it on or changing the volume)?

f) Were there any unexpected reactions or side effects?

g) What aspects of the device did you like?

h) What aspects of the device would you change if you could?

4. Exploring your experience with the EaseVRx+ software:

a) What are your general thoughts and feelings about the program?

b) Who do you think is the target user that was considered when the program was created?

c) How easy or difficult was it to use the program?

- What did you think about navigating the menus?

d) Discuss your experience with the gaze-based selection.

- Did you switch to controller use?

- What prompted a switch, if any, and how did that affect your experience?

e) Describe the experiences within the software.

- Did something personal or from your culture make certain things stand out more?

f) Talk about the elements that you think the developers got right.

g) What advice would you give the developers that could make the experience better for you and other people in your community?

5. Reflect on how your relationship with the VR headset evolved over time.

- What events might have influenced this shift?

6. Considering your background and what you've been through, how did these experiences connect with your time in VR?

7. Were there elements of the VR content that you felt were more or less engaging based on your cultural or social background?

8. Imagine people in your neighborhood using this technology. What about the technology might make it easy or hard for them?

9. If you were not enrolled in the study, how accessible would VR be for you?
